# Methods for Evaluating Emotions Evoked by Food Experiences: A Literature Review

**DOI:** 10.3389/fpsyg.2018.00911

**Published:** 2018-06-08

**Authors:** Daisuke Kaneko, Alexander Toet, Anne-Marie Brouwer, Victor Kallen, Jan B. F. van Erp

**Affiliations:** ^1^Kikkoman Europe R&D Laboratory B.V., Wageningen, Netherlands; ^2^Microbiology and Systems Biology, TNO, Zeist, Netherlands; ^3^Perceptual and Cognitive Systems, TNO, Soesterberg, Netherlands; ^4^Human Media Interaction, University of Twente, Enschede, Netherlands

**Keywords:** food-evoked emotion, physiological, behavioral, cognitive, toolbox, emotional processing level

## Abstract

Besides sensory characteristics of food, food-evoked emotion is a crucial factor in predicting consumer's food preference and therefore in developing new products. Many measures have been developed to assess food-evoked emotions. The aim of this literature review is (i) to give an exhaustive overview of measures used in current research and (ii) to categorize these methods along measurement level (physiological, behavioral, and cognitive) and emotional processing level (unconscious sensory, perceptual/early cognitive, and conscious/decision making) level. This 3 × 3 categorization may help researchers to compile a set of complementary measures (“toolbox”) for their studies. We included 101 peer-reviewed articles that evaluate consumer's emotions and were published between 1997 and 2016, providing us with 59 different measures. More than 60% of these measures are based on self-reported, subjective ratings and questionnaires (cognitive measurement level) and assess the conscious/decision-making level of emotional processing. This multitude of measures and their overrepresentation in a single category hinders the comparison of results across studies and building a complete multi-faceted picture of food-evoked emotions. We recommend (1) to use widely applied, validated measures only, (2) to refrain from using (highly correlated) measures from the same category but use measures from different categories instead, preferably covering all three emotional processing levels, and (3) to acquire and share simultaneously collected physiological, behavioral, and cognitive datasets to improve the predictive power of food choice and other models.

## Introduction

People experience and appreciate many types of food and beverages (referred to as “foods” in this study) during their life. Specific emotions have been considered as determinants of affective responses to foods (Ferber and Cabanac, 1987; Willner and Healy, 1994) and food-related behavior including food choice (e.g., Oliver and Wardle, 1999; Russell, 2003; Gibson, 2006; Thomson et al., 2010; Ng et al., 2013; Dalenberg et al., 2014; Gutjar et al., 2015a; Kenney and Adhikari, 2016). Liking ratings do not predict food choice behavior accurately (Zandstra and El-Deredy, 2011; Griffioen-Roose et al., 2013). Gutjar et al. (2015b) suggested that food-evoked emotions can predict individual's food choice more accurate than liking scores. Dalenberg et al. (2014) mention that consumers' emotions add predictive power to a food choice (predicting) model based on hedonic scales. These studies suggest that assessing emotional responses to foods may reveal previously unknown product attributes which can be a valuable source of information for product development and marketing that goes beyond traditional sensory and acceptability measurements (Thomson et al., 2010). Therefore, it is important to obtain valid and reliable (combinations of) measurements of food-evoked emotion.

Despite its importance, different authors use different definitions of emotion. For instance, King and Meiselman (2010) define emotions as brief, intense physiological and mental reactions, Gibson (2006) defines emotions as short-term affective responses to the appraisal of particular stimuli, Bagozzi et al. (1999) define emotion as a mental state of readiness that arises from cognitive appraisals of events or thoughts, and Cabanac (2002) proposed that an emotion is any mental experience with high intensity and high hedonic content (pleasure/displeasure). These different definitions contain elements of both internal and external, bottom-up and top-down, as well as physiological and cognitive elements. All these facets are considered relevant and illustrate that there is not a single measure that would be able to capture the full range of relevant aspects. To organize the complex response patterns, we introduce a conceptual framework in section Assessment Methods and Their Classification, including the methodologies to assess the different response patterns as there is a wide variety of instruments available.

Verbal self-reporting questionnaires are the most commonly used techniques to measure emotional responses, due to their ease of application, cost-effectiveness and discriminative power (Churchill and Behan, 2010; Dorado et al., 2016). However, they have specific shortcomings, including: (1) emotions are difficult to verbalize (Köster and Mojet, 2015), (2) the “emotional” lexicon varies across cultures and languages, particularly when it comes to foods (Gutjar et al., 2015b), (3) verbalizing emotions can interfere with the food experience itself, and (4) self-reports only capture conscious, declared opinions (Winkielman et al., 2011; Venkatraman et al., 2015). Wilson et al. (1993) asked their participants to answer whether they liked or disliked five different posters with or without providing the reason why. Subsequently, they could take one of the posters with them. Participants who provided the reasons were less satisfied with their choice 3 weeks later (Wilson et al., 1993) showing that questioning individuals about affective experience can affect the affective experience itself. Regarding EsSense Profile, one of the most widely used self-report questionnaires for evaluating an individual's emotional responses (King and Meiselman, 2010), Jaeger et al. (2013) stated that this technique might not capture the full range of emotions individuals may experience in response to food and therefore may not properly measure food-evoked emotions. Thus, it seems worth the effort to include other types of measurements as well like behavioral and physiological measurements. Köster (2009) proposed that research groups should develop implicit measurement techniques and use these where possible and combine them with explicit measures if feasible in order to compare and eventually cross-validate results Examples include facial expression recognition (happy, sad, angry, surprised, scared, and disgust: Kostyra et al., 2016) and physiological variables reflecting activity of the autonomic nervous system (ANS: de Wijk et al., 2012). However, there is no “golden standard” to assess food-evoked emotions at this moment yet.

We aim to provide an exhaustive list of tools that have been used to measure food-evoked emotions over the last 20 years. We also categorize them using a general model describing the relevant aspects of emotion processing and the range of methodologies to assess the relevant facets (see section Assessment Methods and Their Classification). This categorization helps to identify gaps in the currently prevailing set of instruments and enables researchers to choose (a combination of) measures in a balanced way. Our categorization indicates to what extent different methods are redundant or complementary and helps researchers in this area to compile a set of complementary methods that provides the maximal amount of information. In addition, it may serve to guide further development of new methods to assess food evoked emotions that predict future consumer behavior.

## Literature search

We used the databases of PsycINFO to select relevant articles that were published between January 1997 until the end of December 2016.

### Inclusion criteria

We used the following inclusion criteria:

1. The article should report empirical studies in peer-reviewed journals and be written in English.
2. The article should include original data from healthy human populations.
3. The study should investigate consumers' emotions evoked by directly experiencing foods. “Direct experience” could be tasting foods, viewing images of food, or sniffing food odorants. The following were considered to be indirect (and therefore excluded from this review): viewing packages, viewing printed names of brands and thinking about food or beverages (e.g., by asking “*How do you feel when you think of “apple”?*”).
4. In this study the term “emotions” includes hedonic liking, pleasantness, preference, and moods.

Gibson (2006) describes moods as more long-lasting psychological arousal states than emotions with interacting dimensions related to energy, tension and pleasure that may appear and persist in the absence of obvious stimuli and may be more covert to observers. However, mood and emotion both reflect emotional states and are often used interchangeably in common language (Köster and Mojet, 2015). Also, as Gibson (2006) mentions, the relationships between mood, emotions and physiological arousal may be complex. Therefore, we included “mood” in our criteria. While the sensory characteristics of food (e.g., appearance, aroma, taste and texture) are important drivers of emotional experience, we here focus on methods to measure food-evoked emotion, and not on methods to assess the perception of sensory characteristics (e.g., this tastes sweeter and feels softer than others). Thus, studies on the appraisal of the qualitative characteristics of foods (e.g., intensity of sweetness, sourness, saltiness, spiciness, and bitterness) are not included here.

### Search procedure

**Figure 1:**
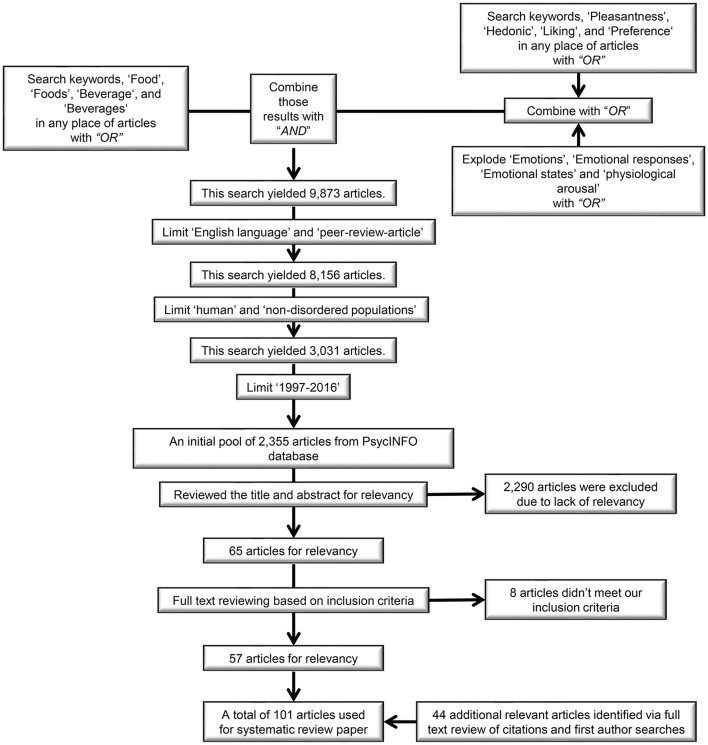
Schematic representation of literature search and selection procedure.

Three reviewers (DK, AT, and AB) constructed the inclusion criteria, searched and evaluated the relevant literature. To obtain relevant articles from the PsycINFO database, the following combination of keywords was used: (food *OR* foods *OR* beverage *OR* beverages) *AND* [(“explode” emotions *OR* emotional responses *OR* emotional states *OR* physiological arousal) *OR* (pleasantness *OR* hedonic *OR* liking *OR* preference)]. We used the “explode” function in the PsycINFO search tool. For instance, exploding “emotions” provides all articles related to emotions. In addition to exploding emotion-related keywords, we further searched relevant articles using keywords such as pleasantness, hedonic, liking, and preference. As a result, we obtained an initial pool of 9,873 articles. Then, by limiting our search to articles in English reporting empirical studies in peer-reviewed journals, 8,156 articles were selected. Among them, we excluded articles targeting animals and disordered populations and kept 3,031 articles. We finally obtained 2,355 articles published in a 20 year period ranging from 1997 to 2016 (Table S1). Based on reviewing the title and abstract of those articles, we excluded articles with a lack of relevance (i.e., they did not meet our inclusion criteria as described above) and ended up with 65 articles. Then, full-text screening resulted in 57 relevant articles. For most of the relevant papers, measuring food-evoked emotions was not the main topic but part of the methodology to answer a different question of interest. This is why it proved difficult to capture *all* relevant articles using keywords like the ones listed above. Another 44 relevant articles were extracted based on cited references in the set of 57 articles and based on searches for more work by the first author, resulting in a final set of 101 papers. The eligibility of these additional 44 articles was independently assessed and confirmed by all three reviewers via an in-depth critical full-text review. A schematic representation of the search procedure is shown in Figure 1.

### Overview of selected articles

**Figure 2:**
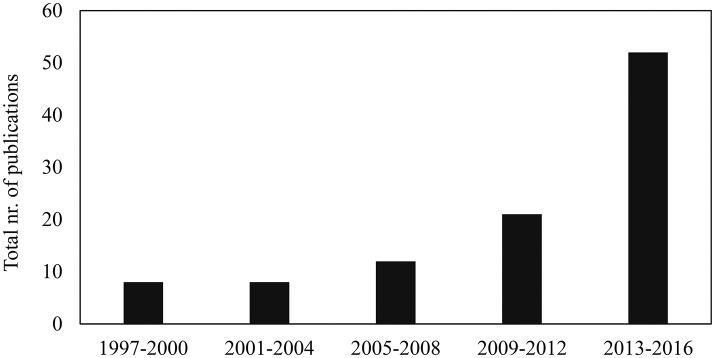
Total number of publications over successive 4-year intervals from 1997 to 2016.

**Figure 3:**
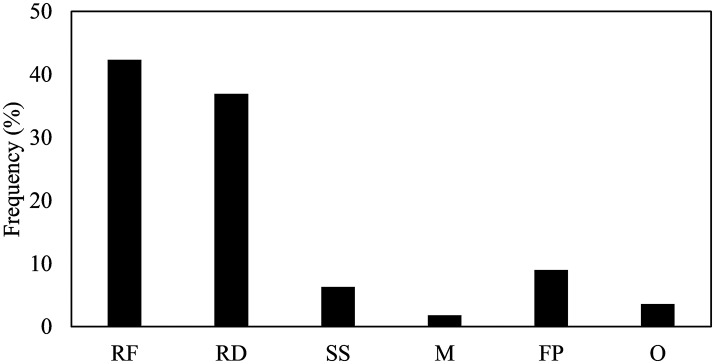
The frequency of stimuli used within 101 studies (RF, Regular solid foods; RD, Regular drinks; SS, Simple solutions; M, Meal; FP, Food Pictures; O, Odors).

Table S2 gives a summary of the final set of 101 articles about the stimuli, the methods to measure food-evoked emotions, and the key findings provided by those methods. More than half of these articles were published in the last 4 years, and about ten times more articles were published in the last 4 years than during the first 4 years (Figure 2). This suggests that there is a growing interest in understanding emotions evoked by foods. Figure 3 gives an overview of the stimuli that have been used to evaluate food-related emotions. Actual foods were used by far most often as stimuli. Representative foods stimuli were sweet products, such as chocolates and cakes, while savory foods were less frequently selected as stimuli. Most studies evaluated an individual's emotions for a sole product, not for a full meal. The vast majority of measures were conducted just before, during and right after experiencing foods, although some studies asked participants to report their emotions a certain amount of time after experiencing the sample stimuli.

**Figure 4:**
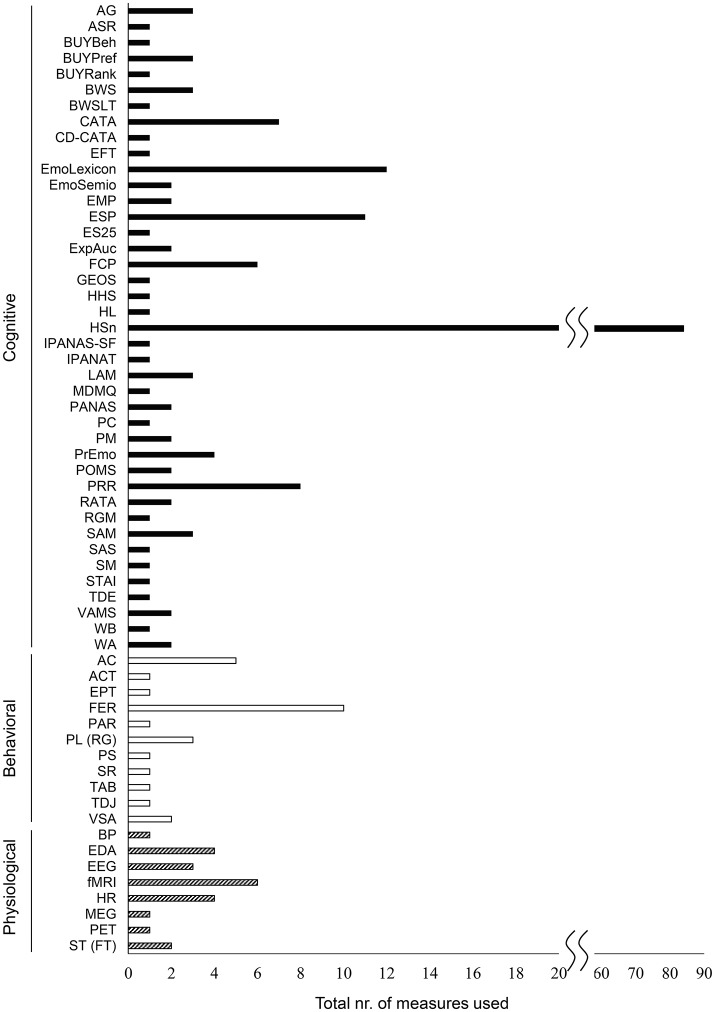
Total number of studies (from the selected set of 101) in which each of the measures is used. The black bars, the white bars, and the shaded bars represent the cognitive, behavioral, and physiological measures, respectively. (All abbreviations are described in Table 1).

From the 101 selected articles, we identified a total of 59 different measures for the assessment of food-evoked emotions. Table 1 presents a brief description of each of these 59 different measures and a reference to a more elaborate description. The total number of times each measure was used within the selected group of 101 relevant studies is depicted in Figure 4. In this figure, we also grouped the measures according to general type of methodology (physiological, behavioral and cognitive). More than 80% of the papers used hedonic scaling measures to evaluate food-evoked emotions, indicating that this measure is most often used in addition to other measures. Following hedonic scales are several versions of emotional lexicon questionnaires such as CD-CATA, EsSense Profile, and unique instruments created by researchers themselves. Recording facial expression, usually by analyzing picture or movie data or electromyography (EMG), is a popular behavioral/implicit method. It should also be noted that more than 50% of measures were only used once among the 59 measures extracted from the 101 selected articles, indicating that there is still no representative measure or combination of measures developed for the evaluation of food-evoked emotions.

**Table 1:** Brief description and abbreviation of measures used for the assessment of food elicited emotions.

**Method**	**Abbreviation**	**Description**	**References**
Amount consumed	AC	The weight, volume or number of food or drink products that are consumed. This measure tends to increase with hedonic evaluation.	Zandstra et al., 1999
Autobiographical congruency test	ACT	The ease and speed with which people remember sad or cheerful events in their lives are higher when they are congruent with their present emotional state.	Mojet et al., 2015
Affect grid	AG	Two-dimensional scale to assess affect along the dimensions pleasure and arousal.	Russell et al., 1989
Affect self report scale	ASR	A self-report scale with 18 affective terms that can be scored on 7-point scales.	Christie and Friedman, 2004
Blood pressure response	BP	Blood pressure is the pressure of circulating blood on the walls of blood vessels, usually expressed in terms of the systolic (maximum during one heart beat) pressure over diastolic (minimum in between two heart beats) pressure, and measured in millimeters of mercury (mmHg), above the surrounding atmospheric pressure. Blood pressure may change in response to changes in mood or emotions.	Bercea, 2013
Buying behavior	BUYB	Actual buying behavior (buying frequency).	Rosas-Nexticapa et al., 2005
Buying preference rating	BUYP	Self-reported likelihood to buy a product.	Rosas-Nexticapa et al., 2005
Buying preference ranking	BUYR	Ranking different products according to self-estimated likelihood to buy.	Rosas-Nexticapa et al., 2005
Best-worst scaling or maximum difference scaling	BWS	Assessors are presented a series of sample triads or tetrads from which they select the (best and worst) samples representing the largest difference in an underlying continuum, e.g., liking.	Jaeger et al., 2008
Best-worst scaling of lexicon terms	BWSLT	For different combinations of 5 words from a larger lexicon, choose which word most/least closely reflects product experience.	Thomson et al., 2010
Check-all-that-apply	CATA	Assessors are presented with a list of sensory terms or phrases and are asked to select all those terms or phrases they consider applicable for describing the focal sample.	Adams et al., 2007
Consumer-defined check-all-that-apply	CD-CATA	Assessors are presented with a list of 36 emotion terms elicited from interviews with consumers and are asked to select all those terms they consider applicable for describing the focal sample.	Ng et al., 2013
Electrodermal activity	EDA	Electrodermal activity measures changes in the electrical resistance of the skin which reflects activation of the sweat gland in reaction to emotional stimuli. EDA is the generic term for all types of skin conductance variables such as skin conductance response (GSR) and skin conductance level (SCL).	Kreibig, 2010
Electroencephalography	EEG	An electrophysiological monitoring method to record electrical activity of the brain. It is typically noninvasive, using electrodes placed along the scalp. EEG measures voltage fluctuations resulting from ionic current within the neurons of the brain.	Bercea, 2013; Agarwal and Xavier, 2015
Empathic food test	EFT	Assessors report their feelings after food consumption using a list of 12 empathic terms rated on 5-point scales.	Geier et al., 2016
EmoSemio	EmoSemio	23 semantic product-specific sentences (16 positive and 7 negative emotions)	Spinelli et al., 2014
EmoSensory profile	EMP	List of 14-17 emotion terms and 13 sensory terms.	Schouteten et al., 2015
Emotive projection test	EPT	Given that people tend to project their feelings onto others, emotions can indirectly be measured from judgments on personality traits of portraits of others.	Mojet et al., 2015
EsSense profile	ESP	List of 39 emotion terms that can either be rated on 5-pt scales (EPRAT) or selected (EPCATA).	King and Meiselman, 2010
EsSense25	EsSense25	A shortened version of the EsSense Profile with 39 emotion terms.	Nestrud et al., 2016
Experimental auction	ExpAuc	The amount of money participants bid in an auction procedure measures their willingness to pay for a certain product.	Poole et al., 2007
Free choice profiling	FCP	Assessors describe products in their own words and rate the perceived intensity of those terms.	Oreskovich et al., 1991
Facial expression response	FER	A facial expression is one or more motions or positions of the muscles beneath the skin of the face. Facial expressions can reflect the emotional state of an individual in response to a stimulus.	Bercea, 2013
Functional magnetic resonance imaging	fMRI	Functional magnetic resonance imaging is a technique for measuring brain activity by detecting changes in blood oxygenation and flow that occur in response to neural activity.	O'doherty et al., 2001
Geneva emotion and odor scale	GEOS	A list with 6 scales (pleasantness, unpleasantness, sensuality, relaxation, refreshment, sensory pleasure) and 36 terms developed to investigate odor-elicited affective feelings.	Chrea et al., 2009
Hybrid hedonic scale	HHS	A linear scale with marked equidistant points and verbal affective labels serving as anchors in the middle and extreme regions of the scale.	Villanueva et al., 2005
Hard laddering	HL	Using an a priori defined list of paired products in combination with a structured questionnaire, assessors are asked to indicate their choice priority, and to provide arguments for their choice. (Note: In Soft Laddering the participant is interviewed by a trained experimenter.)	Russell et al., 2004
Heart rate response	HR	Heart rate is the speed of the heartbeat measured by the number of contractions of the heart per minute (bpm). Changes in heart rate can reflect changes in the state of arousal of an individual.	Bercea, 2013
n-point Hedonic Scale (hedonic rating)	HSn	Hedonic, pleasantness or liking scales typically use (5, 7 or 9 point) category scales and uni- or bipolar magnitude estimation scales to give numerical estimates of liking.	Lim, 2011
International positive and negative affect schedule (PANAS) short form	I-PANAS-SF	Instrument for self-assessment of affect by rating 5 positive and 5 negative emotion terms on 5-point scales. The I-PANAS-SF is a shortened version of the PANAS.	Thompson, 2007
Implicit positive and negative affect test	IPANAT	Assessors rate to what extent artificial words (e.g., SAFME) fit with three positive (happy, cheerful, energetic) and three negative (helpless, tense, inhibited) emotions.	Quirin et al., 2009
Labeled affective magnitude (LAM) scale	LAM	9-pt hedonic scale with magnitude-scaled semantic labels.	Schutz and Cardello, 2001
Multi-dimensional mood questionnaire	MDMQ	List of 24 (long form) or 12 (short form) items covering 3 bipolar dimensions of mood (i.e., good mood-bad mood, alertness-fatigue, ease-unease).	Geier et al., 2016
Magneto-encephalography	MEG	Magneto-encephalography is a functional neuroimaging technique for mapping brain activity by recording magnetic fields produced by electrical currents occurring naturally in the brain, using very sensitive magnetometers.	Tsourides et al., 2016
Positive and negative affect schedule	PANAS	Instrument for self-assessment of affect by rating 10 positive and 10 negative emotion terms on 5-point scales.	Watson et al., 1988
Postauricular reflex	PAR	The postauricular reflex is a vestigial muscle response in humans that acts to pull the ear backward and that increases with emotional valence.	Hebert et al., 2015
Product choice	PC	Assessors are presented with different products and asked to select the one they prefer for consumption.	Lévy and Köster, 1999
Positron emission tomography	PET	A functional imaging technique that is used to observe metabolic processes in the body.	Bercea, 2013
Pick-up latency	PL	Pick-up latency is based on the principles of approach-avoidance motivations: people are quicker to approach stimuli of positive valence than stimuli of negative valence.	Davies et al., 2012
Preference mapping	PM	Preference mapping is a generic term involving a collection of techniques used to relate detailed sensory profiling data to consumer liking.	Clark, 1998
Profile of mood states	POMS	Psychological 5-point rating scale that can be used to self-assess mood states along 6 different dimensions (Tension or Anxiety, Anger or Hostility, Vigor or Activity, Fatigue or Inertia, Depression or Dejection, Confusion or Bewilderment).	McNair et al., 1971
Product emotion measurement instrument	PrEmo (PrEmo2)	PrEmo is a non-verbal self-report instrument to measure different emotions (visualized by an animated cartoon character) on a 5-point scale.	Desmet et al., 2000Laurans and Desmet, 2012
Rank rating or positional relative rating	PRR	The assessor is given all products at once and orders (ranks) them along a line in order of liking (hedonic order).	Kim and O'mahony, 1998
Postural sway	PS	Hedonic evaluation activates approach vs. avoidance mechanisms that modulate postural sway such that pleasant stimuli elicit anterior-going sway, and unpleasant stimuli elicit posterior-going sway.	Brunyé et al., 2013
Rate-all-that-apply	RATA	Assessors are presented with a list of sensory terms or questions and are asked to rate all those they consider applicable for describing the focal sample.	Ares et al., 2014
Repertory grid method	RGM	Assessors are presented 3 stimuli, first divide them in 2 similar stimuli and 1 different one, and then describe the differences between the products.	Kelly, 2003
Self-assessment mannikin	SAM	Validated 9-point pictorial (anthropomorphic) rating scale for measuring pleasure, arousal and dominance.	Bradley and Lang, 1994
ScentMove	SM	ScentMove is a simplified version of the GEOS and consists of 6 scales (sensuality, relaxation, well-being, energy, nostalgia, disgust) with 3 terms each.	Porcherot et al., 2010
Startle response	SR	The startle response is a largely unconscious defensive response to sudden or threatening stimuli, such as sudden noise or sharp movement, and is associated with negative affect and a state of arousal. The onset of the startle response is a brainstem reflectory reaction (startle reflex) that serves to protect vulnerable parts, such as the back of the neck (whole-body startle) and the eyes (eyeblink).	Koller and Walla, 2015
Skin temperature response	ST	Local skin temperature changes reflect variations in blood flow in response to emotional stimuli.	Kreibig, 2010
State-trait anxiety inventory	STAI	Introspective psychological inventory consisting of 40 self-report items pertaining to anxiety affect.	Spielberger, 1983
Take away behavior	TAB	Assessors are asked to take away any samples tested after experiments without any description.	Wichchukit and O'mahony, 2010
Temporal dominance of emotions	TDE	Assessors periodically check the most dominant out of 10 emotions over the duration of the evaluation process.	Jager et al., 2014
Temporal duration judgment	TDJ	Time is underestimated when looking at pictures of food (compared to neutral pictures), and more so for disliked than for liked foods.	Gil et al., 2009
Visual analog mood scales	VAMS	The Visual Analog Mood Scales is designed to measure 8 different general mood states (sad, happy, tense, anxious, confused, tired, energetic, irritated) on visual analog scales.	Bond and Lader, 1974
Visual selective attention	VSA	The allocation of visual selective attention indicated by eye fixation reflects relative preference for different products (liking).	Bercea, 2013
Word association	WA	Assessors are asked to write down the first images, associations, thoughts or feelings that come to mind.	Schmitt, 1998

## Assessment methods and their classification

To structure the measures described in the set of 101 papers we use a 3 × 3 framework consisting of three levels of emotional processing and three levels of measurement level as described in the next section.

As the first dimension of our 3 × 3 framework (processing level), we use three levels of processing: the lower level referring to unconscious and basic sensory processing, the intermediate level, referring to perception and early cognitive processing, and the higher level, referring to the conscious processing stage after cognition including decision-making and food-related behavior. This first dimension is similar to processing levels as used in SOR (Stimulus-Organism-Response) paradigm introduced by Mehrabian and Russell (1974) and adjusted by Bitner (1992), Lin (2004), and Schreuder et al. (2016). The second dimension of our 3 × 3 framework is the measurement level: physiological, behavioral, and cognitive. Physiological measures (like heart rate and skin conductivity) reflect the (largely unconscious) activity of the autonomic nervous system and bodily functions. Behavioral measures (like face and body movement and choice reaction time) reflect the unconscious and conscious responses of the body. Finally, the cognitive measures (like rating scales and questionnaires) reflect conscious opinions, choices, and decisions. This 3 × 3 framework provides a concise description of the different processing levels involved in the experience of multisensory environmental stimuli and their link to perceptual, emotional, and cognitive and behavioral outcomes. This framework is, therefore, well suited for our purposes.

Below, we discuss each of the nine combinations of processing and measurement levels in regard to assessing food-evoked emotions and used these nine categories to classify the 59 different measurement instruments reported.

### Lower processing level (sensory processing)

When presented with food stimuli, individuals perceive and integrate information from all senses: vision, audition, taste, olfaction, and touch through unconscious, neurophysiological processes. Measures used to evaluate the emotional aspects of these processes were grouped in this category.

#### Physiological measures

Major peripheral physiological measures like heart rate (HR), electrodermal activity (EDA), skin temperature (ST), and blood pressure (BP) fall into this measurement level. HR is a cardiovascular measure and the most frequently used measure to evaluate emotional states as e.g., induced by viewing emotional pictures or film clips (Kreibig, 2010). However, HR has not been used much to evaluate emotions evoked by experiencing foods. One of the exceptions is a study conducted by de Wijk et al. (2012), who demonstrated that HR can indeed be used to assess food-related emotions resulting from the sight, smell, and taste of liked and disliked foods. Similar to HR, EDA has often been used to investigate how people react to viewing emotional pictures and film clips. The study by de Wijk et al. (2012) included EDA as well. Skin Temperature (ST, also referred to as finger temperature or FT) is a measure reflecting autonomic nervous system activity. Rimm-Kaufman and Kagan (1996) suggested that researchers interested in emotion might consider using ST as an informative variable recorded with infrared tele thermography. Similar to HR, Blood Pressure (BP) is also used to examine an individuals' unconscious emotional states (Kreibig, 2010). Marczinski et al. (2014) used BP and found that the consumption of energy drinks elevated BP, while it is still unclear which part of this rise was caused by the intake of nutrients and which part was caused by emotion.

In addition to these peripheral physiological measures, measures reflecting brain activity such as electroencephalography (EEG), magnetoencephalography (MEG), functional magnetic resonance imaging (fMRI), and positron emission tomography (PET) can be also used to evaluate an individual's unconscious response to food stimuli at the physiological level (Bercea, 2013; Agarwal and Xavier, 2015). Event-related potentials (ERPs) are specific positive or negative peaks in the EEG following the presentation of a stimulus. An example is the P300, the size of which relates to the amount of attention given to the stimulus (Hoffman and Polich, 1998; Patel and Azzam, 2005). In addition to the P300, the late positive potential (LPP) is an ERP component that is related to stimulus control and the use of attentional resources and regulatory factors in the brain (Hajcak et al., 2009). MEG is a functional neuroimaging technique that maps electrophysiological activities inside the brain through very sensitive magnetometers (Yoshikawa et al., 2014; Tsourides et al., 2016). For instance, MEG identified a robust neural correlate of the food and non-food distinction (Tsourides et al., 2016). fMRI is another technique to evaluate more detailed activities and responses inside the brain by detecting changes in blood oxygenation and flow that occur in response to neural activity. For instance, Grabenhorst et al. (2008) showed that perceived pleasantness correlated with activity in the orbitofrontal cortex (OFC) and the pregenual cingulate cortex. More recently, Hoogeveen et al. (2015) demonstrated that older people reported higher liking ratings for sweet and salty, lower ratings for sour, and similar ratings for bitter compared to young people. Their findings indicated that these differences between younger and older adults may be associated with the reduction of right amygdala activity in older persons. PET is a functional imaging technique that is used to observe metabolic processes. Small et al. (2001) combined PET and MRI and demonstrated that different neural substrates mediate positive or appetitive and negative or aversive stimuli.

It is important to note that for every physiological sensor, several categories of physiological variables can be extracted (e.g., heart rate variability (HRV) and HR for ECG, and ERPs and power spectra for EEG). Within these categories there are usually further differences as to how the variable is defined. For instance, from subsequent RR intervals, HRV can be defined as the Root Mean Square of the Successive Differences (RMSSD), or as the power in frequency bands of interest (Veltman and Gaillard, 1998). In addition, extracting these indices relies heavily on choices with respect to time intervals across which data is examined and advanced data-processing techniques to filter, clean and classify the, often noisy, data from the physiological sensors. When using advanced analyses such as deep learning, it is not uncommon to try and compare different algorithms or parameter settings (e.g., Saeed et al., 2017). Finally, we want to point out the fact that physiological variables can be affected by body movements or time related factors such that failing to properly control or correct for those could lead to incorrect conclusions (Brouwer et al., 2015). These aspects need to be kept in mind when reviewing and comparing studies using physiological measures. A detailed description and discussion of the different signal processing techniques as used in the studies using physiological measures referred to in this review is outside the scope, but literatures exist on good practice [e.g., for machine learning in the context of EEG (Lotte et al., 2007); for heart rate variability (Camm et al., 1996)].

#### Behavioral measures

A representative measure here is startle response (SR), also known as the alarm reaction or the startle reflex. The SR is a completely natural, involuntary reaction to a stimulus such as a flash of light, a sudden threatening movement or loud noise, and is associated with negative affect. Walla et al. (2010) provided evidence that SR modulation (eye blinks in their study) can be used reliably to quantify human motivational states related to the intake of different kinds of food (Walla et al., 2010). The postauricular reflex (PAR) is a vestigial muscle response in humans that acts to pull the ear backward and can be also grouped into this category. A study by Hebert et al. (2015) suggests that both PAR and SR are modulated by emotional states with valence scores of appetitive, neutral, and disgusting food images affecting SR in a direction opposite to PAR. In particular, pleasant stimuli enhanced the PAR (Gable and Harmon-Jones, 2009). Sandt et al. (2009) suggest that the PAR might be useful to measure appetitive responding in human. Another unconscious behavioral index categorized in this group is the postural sway (PS): a covert horizontal movement in response to a stimulus. There is strong evidence for bidirectional links between approach and avoidance (i.e., motivational state) and overt and covert indices of motor behavior (Elliot and Covington, 2001), including PS. Brunyé et al. (2013), using 100 food images, found evidence that individual preferences modulated anterior–posterior postural sway, with pleasant stimuli eliciting anterior-going sway and unpleasant stimuli elicit posterior-going sway.

#### Cognitive measures

The lower emotional processing level is defined as the stage in which stimuli are automatically and unconsciously processed through our senses and the brain's sensory cortices without conscious intervention or interpretation. Cognitive measures rely on for instance individuals rating their food-evoked emotions and are thus not applicable at this level of processing.

### Intermediate processing level (perception and early cognitive processing)

Following the integrated multisensory perception of food stimuli, individuals relate their percepts to previous experiences and information stored in memory. This can occur through both conscious and unconscious processes. This intermediate processing level concerns a short-term emotional state that is directly related to the object of focus. This state can be observed consciously (feeling aroused, pleasant, etc.) or can be experienced unconsciously, and drives the allocation of processing resources and priorities for the consecutive processing level (cognition, behavior, and decision).

#### Physiological measures

Frontal alpha asymmetry measured using EEG fits in this category. Some studies, using pictures of desserts, showed that alpha asymmetry is an unconscious response that depended on whether the subject would like to approach or avoid that dessert (Gable and Harmon-Jones, 2008; Harmon-Jones and Gable, 2009). Such a response can only occur after the perception of the dessert has been integrated with information from memory. Other neuroimaging techniques (fMRI, MEG, and PET) were also considered as measures to assess the intermediate processing level.

#### Behavioral measures

The autobiographical congruency test (ACT) and the emotive projection test (EPT) measure the reaction time needed to think of a happy or a sad life event and are employed to indirectly measure food-related emotions (Mojet et al., 2015). Mojet et al. (2015) showed that the ACT didn't differentiate between products, and that the EPT was the most promising measure since it had no significant correlation with either liking and differentiated between products. The temporal duration judgment (TDJ) evaluates how long someone is looking at food images. Gil et al. (2009) provided evidence that the time looked at disliked food images was longer and at liked food shorter than the time looked at neutral food images. The pick-up latency (PL) method is another indirect measure for liking based on the principles of approach-avoidance motivations: PL is smaller for positive valence and larger for negative valence (Krieglmeyer et al., 2010). Davies et al. (2012) showed that PL was reduced for positively conditioned flavors and increased for negatively conditioned flavors. Visual selective attention (VSA) is a related behavioral measure: a transitory decline in the pleasantness of the taste modulates covert VSA (di Pellegrino et al., 2011). Finally, the facial expression response (FER) resulting from the integrated stimulation evoked by food experience over a short time period is a behavioral measure to assess the intermediate processing level. Several recent studies provide evidence that the FER correlates with valence and arousal ratings (de Wijk et al., 2012, 2014; Garcia-Burgos and Zamora, 2013; Danner et al., 2014; He et al., 2016).

#### Cognitive measures

This category contains instruments that do not rely on directly asking questions about the subject's emotions (as these would tap into the higher emotional processing level) but on implicit cognitions. The experimental auction (ExpAuc) technique is such an instrument, using a real product and real money (Poole et al., 2007). They showed that the ExpAuc implicitly measures an individual's willingness to pay for a certain product. A second instrument in this category is the implicit association test (IAT: Greenwald et al., 1998): a tool to measure implicit attitudes toward stimuli. However, no study using an IAT on food stimuli was present in our final set of articles.

### Higher processing level (conscious reflection and decision making)

The higher processing level could be considered as the final stage in which individuals consciously recognize what foods are, which emotions they evoke, how these are associated with their social relationships, how food stimuli are related with their expectations, etc. The measures that evaluate these conscious emotions were categorized in this group.

#### Physiological measures

All of the physiological responses to food stimuli are unconscious and automatically occur in the human body and brain. Because of the implicit assumption that individuals are not able to intentionally control their physiological response to food stimuli, none of the papers selected for this review employs a measure at the physiological level to assess the higher level of emotional processing.

#### Behavioral measures

While the behavioral measures at the intermediate processing level reflect unconscious, short-term (immediate) emotional state, the instruments at the higher level relate to more deliberate approach or avoidance behaviors, influenced by more cognitive and long-term emotion. In this category, the measures of Amount Consumed (AC: Zandstra et al., 1999) and Take Away Behavior (TAB: Weiss et al., 2010; Wichchukit and O'mahony, 2010) can be included.

#### Cognitive measures

There are many instruments that fall within this category (more than in all eight other categories combined). Therefore, we use five subcategories to provide further structure: (1) hedonic scaling, and questionnaires with (2) preferable foods, (3) emotional lexicons, (4) emotional pictures, and (5) mood-related lexicons.

##### Hedonic scaling

In Hedonic Scaling using a *n*-point scale (HSn), product evaluation (liking) is typically scored on 5-point (Brunyé et al., 2013), 7-point (Caporale et al., 2009; Awazu, 2013) or 9-point (Ares et al., 2010; Chung et al., 2012; Bhumiratana et al., 2014) liking scale. Adjectives are sometimes used to label the points in order to aid the interpretation. In most studies consumers are asked to rate several samples sequentially without reference to other elements in the set (a serial monadic approach). Some modified methods from HSn are Labeled Affective Magnitude (LAM) scale and Hybrid Hedonic Scale (HHS). Schutz and Cardello (2001) developed the LAM scale, which is 9-point hedonic scale with magnitude-scaled semantic labels. HHS is also a linear scale with marked equidistant points and verbal affective labels serving as anchors in the middle and extreme regions of the scale (Villanueva et al., 2005). Finally, Preference Mapping (PM) is a technique to describe the relationship between hedonic ratings of a randomized population of subjects and the sensory scores of the products rated by trained panels (Clark, 1998).

##### Questionnaires choosing preferable foods

Questionnaires asking participants to select one or more products are also based on their higher processing level of food stimuli. Instruments in this subcategory are the Product Choice (PC) test, where assessors are presented with different products and asked to select the one they prefer for consumption (Lévy and Köster, 1999), the Free Choice Profiling (FCP) test, where assessors describe products in their own words and rate the perceived intensity of those terms (Kim et al., 2013), and the Positional Relative Rating (PRR) test. Kim and O'mahony (1998) used the latter measure, in which assessors are given all products at once and order them along a line in order of liking. The Hard Laddering (HL) method also belongs in this category. In a laddering task, assessors are asked to compare products or their attributes and elicit their reasons for choosing a certain product for purchase or consumption. In HL, a structured questionnaire is applied, while in soft laddering the participant is interviewed by a trained experimenter (Russell et al., 2004). There was no study using the soft laddering technique selected by our inclusion criteria. In addition to using HL and studying their Buying Behavior (BUYBeh), Rosas-Nexticapa et al. (2005) also asked participants to rate their Buying Preference (BUYPref) and Buying Ranking (BUYRank). They demonstrated that these ratings might predict purchase frequency of products over a 1 year period of experiments. However, this type of study is too costly and time-consuming to be practical, and these rating scales do not reflect a person's actual buying behavior in all circumstances as shown by Lange et al. (2002). The last measures in this subcategory are the Best-Worst Scaling (BWS: Jaeger et al., 2008) and the Best-Worst Scaling of Lexicon Terms (BWSLT: Thomson et al., 2010).

##### Questionnaires with emotional lexicons

King and Meiselman (2010) compiled a list of 39 emotional terms that consumers associate with products, known as the EsSense Profile (ESP). Each of these terms is rated on a 5-point scale. When applied to evaluate food products, the ESP provides additional information that is not explained by overall product liking (King et al., 2010). A shortened version of ESP, named EsSense 25 (compiling 25 emotional terms) was developed later (Nestrud et al., 2013). Also, Spinelli et al. (2014) developed a product-specific questionnaire, called EmoSemio, based on one-on-one interviews conducted with a modified version of the Repertory Grid Method (RGM: Kelly, 2003). They provided evidence that EmoSemio discriminated product specific emotions better than ESP with chocolate and hazelnut spreads as samples (Spinelli et al., 2014). The Scent Move (SM: Porcherot et al., 2010) is also a tool using an emotional lexicon (a simplified version of the Geneva Emotion and Odor Scale or GEOS: Porcherot et al., 2010) as is the Check-All-That-Apply (CATA) technique, in which assessors are presented with a list of sensory emotional terms or phrases and are asked to select all those terms or phrases they consider applicable to describe the focal sample (Adams et al., 2007). The modified CATA measure to evaluate food-evoked emotions is the Consumer-Defined Check-All-That-Apply (CD-CATA), and was developed and demonstrated by Ng et al. (2013). The Rate-All-That-Apply (RATA) is a rating-based variant of CATA (Ares et al., 2014). In the Word Association (WA) technique, assessors are asked about concepts, images and thoughts that come into their mind for each product, yielding thoughts and associations about the products, after conscious evaluation. The Temporal Dominance of Emotions (TDE) tool is based on the Temporal Dominance of Sensations (TDS), which evaluates the sequence of dominant sensations of a product during a certain time period (Pineau et al., 2003), but with emotional instead of sensory attributes (Ares et al., 2008). They showed that temporal emotional attitude was related to the sensory profiles obtained with TDS. Other self-reported emotion questionnaires were also included in this category, such as EmoSensory Profile (EMP) rating (a combination of 14–17 emotional terms and 13 sensory terms: Schouteten et al., 2015), Affect Self Report (ASR) scale (rating 18 affective terms on a 7-point scale: Christie and Friedman, 2004), Empathic Food Test (EFT; rating 12 empathic terms: Geier et al., 2016), Positive and Negative Affect Schedule (PANAS; rating 10 positive and 10 negative emotion terms: Watson et al., 1988), Implicit Positive, Negative Affect Test (IPANAT; rating 3-positive and 3-negative emotions: Quirin et al., 2009), and International Positive and Negative Affect Schedule Short Form (I-PANAS-SF: Thompson, 2007), a shortened version of the PANAS (Watson et al., 1988; Thompson, 2007).

##### Questionnaires with emotional pictures

The Self-Assessment Mannikin (SAM) developed by Bradley and Lang (1994) is a measure to evaluate emotions (valence, arousal, and dominance) that uses pictures instead of text (as do the emotional lexicons described above). Similar to the SAM, the Affect Grid (AG: Russell et al., 1989) and PrEmo (PrEmo-2; Desmet et al., 2000; Laurans and Desmet, 2012) were also developed for participants to more easily describe their emotions with pictures. Visually expressed emotions are hypothesized to more closely resemble intuitively experienced emotions (Dalenberg et al., 2014). Evidence for this hypothesis stems from EEG-experiments showing that emotion processing is faster for facial expressions than for emotional words (Schacht and Sommer, 2009; Frühholz et al., 2011; Rellecke et al., 2011).

##### Questionnaires with mood-related lexicons

The final subcategory consists of self-report techniques that evaluate mood, such as the Multi Dimensional Mood Questionnaire (MDMQ) that employs 24 (long form) or 12 (short form) items to cover three bipolar dimensions of mood (Geier et al., 2016), the Profile Of Mood States (POMS) that uses ratings of 6 mood states along with 6 different dimensions (McNair et al., 1971), the State-Trait Anxiety Inventory (STAI) that uses ratings of 40 self-report items pertaining to anxiety affect (Spielberger, 1983), and the Visual Analog Mood Scales (VAMS) that employs ratings of 8 different general mood states (Bond and Lader, 1974).

## Discussion

Emotions are considered to be important drivers of food-related cognitions and behavior like food choice and eating behavior. Indeed, Dalenberg et al. (2014) provided the results indicating that the predicting power of individual's food choice got better by adding the evaluation of food-evoked emotions with liking rating scores. As Köster and Mojet (2015) state, there is no doubt that unconscious emotions can play a role in eating and drinking behavior in a way that is independent of hedonic pleasure as measured by liking. In addition, also mentioned in the introduction, some studies provided the results that liking scaling do not predict individual's actual food choice (Zandstra and El-Deredy, 2011; Griffioen-Roose et al., 2013). Valid, reliable and sensitive instruments that assess food-evoked emotions are therefore valuable for fundamental and applied research and for instance in developing new food products and advocating a healthy lifestyle. A complicating factor in this field is that human emotion is a multifaceted construct linked to physiological, behavioral, and cognitive processes, and we may not assume to find a single measure that covers the full range, while there is a general conviction that all facets are relevant.

We listed, organized, and reviewed the prevailing instruments based on a literature review consisting of 101 peer-reviewed articles published between 1997 and 2016. Our main observations are: (1) There is an overabundance of different measures (about 59 in our set of 101 papers); (2) The majority of these measures assess the cognitive level of emotional processing using subjective ratings or questionnaires (i.e., self-reports are over-represented); (3) Articles that report two or more measures generally use measures that all tap into the same level of emotional processing, while it may be expected that redundant measures have limited added value.

### The overabundance of measures

As also mentioned in the introduction, the fact that about 59 different measures are employed in 101 papers makes it evident that there is not a “golden standard” to assess food-evoked emotions. This has consequences regarding the validity of the measures, the generalizability of reported effects, and the integration (or even meta-analysis) over studies. From a methodological point of view, the use of uncommon measures of which the validity is unknown is not desirable. Some measures are developed and exclusively used by few research groups. For instance, previous studies developed questionnaires with emotional lexicons for a range of product categories (chocolate in Thomson et al., 2010; blackcurrant squashes in Ng et al., 2013; chocolate and hazelnut spreads in Spinelli et al., 2014; coffee in Bhumiratana et al., 2014). Those questionnaires with lexicons are specific for each product and cannot be applied for universal food products. Few other research groups use this technique with the same emotional lexicons. We recommend to choose widely applied, validated measures whenever possible. One should not construct one's own instrument before having verified that there is not an existing tool that may serve one's goals. Both our Tables 1, 2 may be of assistance here.

**Table 2:** The toolbox table: a categorization of all 59 emotional measures extracted from our set of 101 articles.

		**Emotional processing level**		
		**Low level (unconscious, sensory)**	**Intermediate level (perceptual, early cognition)**	**High level (conscious, decision making)**
				
				
	**Physiological**	BP, EDA, EEG (ERPs), fMRI, ST, HR, MEG, PET	EEG (frontal alpha asymmetry), fMRI, MEG, PET	N/A
				
				
	**Behavioral**	PAR, PS, SR	ACT, EPT, FACS, PL, TDJ, VSA	AC, TAB
				
				Hedonic Scaling: HHS, HSn, LAM, PM
**Measurement level**				Questionnaire with preferable foods: BUYB, BUYP, BUYR, BWS, BWSLT, FCP, HL, PC, PRR, SL*, WB
	**Cognitive**	N/A	ExpAuc	Questionnaire with emotional lexicons: ASR, CATA, CD-CATA, EFT, EmoSemio, EMP, ESP, ES25, GEOS, IPANASSF, IPANAT, PANAS, RATA, RGM, SM, TDE, WA
				Questionnaire with emotional pictures: AG, PrEmo, SAM
				Questionnaire with mood-related lexicons: MDMQ, POMS, STAI, VAMS

* *SL (Soft Laddering) were not extracted from our inclusion criteria*.

### The over-representation of self-report measures

Of the 59 measures reported, eight are at the physiological, 11 at the behavioral, and 40 at the cognitive measurement level. More than 60% of the reported measures are based on self-reports assessing cognitive emotional processing. Among them, more than 80% studies used a HSn measure as one of the measures. There are two important comments to be made here. First, as also discussed in the introduction, self-reports (although successful) have inherent shortcomings: emotions are difficult to verbalize, the “emotional” lexicon varies across cultures and languages, answers may be biased, and verbalizing emotions can interfere with food experience itself. Second, self-reports assess almost per definition the higher levels of emotional processing and cannot assess unconscious emotional processing, while this is deemed important to improve both our understanding of food-evoked emotions as well as our algorithms to predict future cognition and behavior. As indicated from the toolbox table with 3 × 3 framework provided here (Table 2), most of the papers selected by our inclusion criteria are of a mono-disciplinary nature and contribute more to the perpetuation of the narrow tunnel view of the cognitive measurement level.

### The limited added value of redundant measures

Although authors often report the data of two or more measures, these are almost always of the same category, again reflecting the over-representation of self-report measures: the majority of those studies used a combination of HSn (including liking and pleasantness scale) ratings and questionnaires with emotional lexicons. These redundant tests with comparable results can be useful to prove the robustness of a specific effect such as hedonic asymmetry. Hedonic asymmetry refers to the finding that people overwhelmingly use positive rather than negative words, whether describing recalled food experiences or describing reactions to food samples. Hedonic asymmetry was described by Desmet and Schifferstein (2008) and has been replicated for different types of commercial products by using ESP and LAM (Cardello et al., 2012), ESP and HSn (King and Meiselman, 2010), CD-CATA and HSn (Ng et al., 2013), and with the original emotional lexicons and HSn (Desmet and Schifferstein, 2008; Manzocco et al., 2013). High correlation between tests was also found by Cordonnier and Delwiche (2008) who reported that PRR yielded similar results to HSn with lemonades as stimuli. Similarly it has been reported that LAM has equal reliability and sensitivity to HSn and a somewhat greater discrimination ability among highly liked foods than HSn (Schutz and Cardello, 2001). These results indicate that, although redundant instruments showing the same results may improve the robustness of the findings, the added value of their repeated use may be limited and the scope of the conclusions is necessarily restricted to that of the chosen measurement category. This limited added value may not outweigh the extra costs of the test and the burden subjected to the participants. In case the redundant instruments show contradictory effects, this could be a useful indication for the lack of robustness and replicability of the effect, but one should carefully consider the quality of the data and the validity of one or both tests. We recommend to refrain from using instruments from the same category. We do recommend using multiple tests but to choose tests from different categories (see below under toolbox for guidance on how to choose your combination of tests, that should preferably be along the diagonal of Table 2). Although these different measures sometimes provided complementary information, there were also many cases in which they yielded redundant and sometimes even contradictory results.

### A toolbox table to support the selection of a combination of instruments

To structure the 59 measures, we used nine categories based on a 3 × 3 framework with the dimensions: measurement level (physiological, behavioral, and cognitive) and emotional processing level (low—unconscious sensory processing, intermediate - perception and early cognitive processing, and high—conscious decision-making and behavior). Furthermore, the category “cognitive high processing level” was divided into five different subcategories. The resulting classification (see Table 2) indicates that current physiological methods are used to evaluate the low and intermediate processing levels, behavioral methods to evaluate all three processing levels, and cognitive methods to evaluate the intermediate and high processing levels. The resulting classification or “toolbox table” (see Table 2) can be used to select a minimal set of methods that provides maximal (complementary) information on the aspects of affective food experience that are of interest, for instance by choosing methods along the table's diagonal from top-left to bottom-right. Current widespread practice is to use one or more measures from the bottom-right category only. Although such measures can certainly provide much information about the consciously experienced effects of food, they may miss essential nuances in feelings and emotions that may influence later behavior. Non-verbal, implicit measurements may complement verbal self-report questionnaires to better understand individual's food-evoked emotions. Although the number of tools in some of the framework's cells is still limited, new instruments have recently been developed and tested. Examples include facial movements, such as smacks of mouth and lips and tongue protrusion for hedonic reactions and gape, eye quench, and nose wrinkle for aversive reactions (Steiner et al., 2001), facial expressions (Kostyra et al., 2016), ANS responses such as HR and EDA as signals for negative emotions like disgust and anger that also provided detailed information on food preference (de Wijk et al., 2012). Using machinelearning techniques, Brouwer et al. (2017) recently found that a combination of different physiological measures showed whether a participant was cooking and tasting a dish with conventional ingredients (a chicken stirfry) or a dish with high arousal, low valence ingredients (a mealworm stirfry).

In addition to these successful demonstrations, some studies conducted a correlation analysis between self-reports questionnaires and physiological or behavioral measurements (Garcia-Burgos and Zamora, 2013; Mojet et al., 2015). Garcia-Burgos and Zamora (2013) conducted linear regression analysis between self-reported hedonic value and the intensity of disgust facial expression analysis on bitter-tasting foods and found a quite low correlation value. The fact that the different categories indeed seem to measure different facets of emotional processing implies that combining them may increase our understanding (for example of the discrepancies between hedonic ratings and consumer choice) and ultimately improve our predictions of food-related cognitions and behavior taking into account that food-evoked emotions is only part of a range of factors that influence future liking, choosing or buying behavior. It was also proposed that the implicit nature of food-related behavior requires the development of more appropriate/adequate research methods that measure the motives of the consumer and her reactions to food in a more implicit way. We recommend acquiring more accumulative and simultaneously collected physiological, behavioral, and self-report datasets.

### Implications for related research

Viewing images of food triggers the desire for the real thing: just looking at pictures of food causes salivation (Spence, 2011) and an uptick in ghrelin, a hormone that causes hunger (Schüssler et al., 2012). These effects increase when images represent food in a more vivid way (Spence, 2011; Moore and Konrath, 2015). Vividness (Steuer, 1992), also referred to as media richness (Daft and Lengel, 1986) refers to the sensory breadth (the number of sensory dimensions) and sensory depth (the information quality and resolution) of stimuli. Vivid stimuli allow observers to fill in more missing sensory information and thereby diminish the user's perception of mediation (i.e., the indirect perception of a product through technical means or devices). This enables users to activate a fuller, more concrete or vivid mental model of a mediated product, which in turn affects their product appraisal (Choi and Taylor, 2014) and intensifies the imagined product experience (Roggeveen et al., 2015). It has for instance been found that vivid (full color) images of pizza elicited higher levels of food craving, a stronger salivation response, and stronger eating intentions, than similar pallid (black and white) images (Moore and Konrath, 2015). Also, vivid food cinemagraphs evoke stronger appetitive responses than similar stills (Toet et al., in press). Virtual reality (VR: Gorini et al., 2010; Nordbo et al., 2015; Ung et al., 2018) and augmented reality (AR: Narumi et al., 2012; Pallavicini et al., 2016) appear to be promising tools to study the impact of environmental cues on human nutritional behavior since they typically provide vivid imagery. In the field of Human-Computer Interaction (HCI), novel multisensory (taste, smell, tactile) interfaces are being developed and used to support studies on food-related emotions and behavior, personal health and wellbeing (Comber et al., 2014; Obrist et al., 2016), or simply to enhance or augment the experience of food (Narumi et al., 2011; Schöning et al., 2012; Spence and Piqueras-Fiszman, 2014; Velasco et al., 2018). HCI can promote healthy food practices and social dining experiences (Comber et al., 2013). The addition of tactile and olfactory channels to VR and AR systems will further enhance the vividness of the mediated perception, and may for instance afford shared distributed virtual multisensory dining experiences (Braun et al., 2016). Multisensory technologies also allow researchers to control the various inputs that accompany a given food experience (Velasco et al., 2018). In all the aforementioned applications it is essential to monitor the emotional and behavioral responses to the perceived virtual or mediated food, and to assess how these responses compare to those evoked by real food (Pallavicini et al., 2016). Reliable emotion assessment techniques are therefore required to further develop and optimize multisensory interactive experiences and to assess the ecological validity of mediated food presentations (Obrist et al., 2016, 2017).

## Conclusions and recommendations

Common practice in assessing food-evoked emotions relies on many different instruments of which the majority only assess a limited aspect of emotional processing. While this common practice resulted in robust and relevant findings, the plethora of different instruments hampers the validation of infrequently used instruments and the comparison of results over studies. The restriction to assess only the cognitive emotional processing level makes it difficult to obtain a complete picture and impoverishes our predictive models. We recommend the following:

(1) use widely applied, validated measures and refrain from constructing a new tool unless absolutely necessary

(2) refrain from using (highly correlated) instruments from the same category

(3) use multiple measures from different categories, preferably covering all three emotional processing levels, for instance by selecting tests along the diagonal of Table 2.

(4) acquire and share simultaneously collected physiological, behavioral, and cognitive datasets to improve the predictive power of food choice and other models.

It must be noted that there are many more factors to fill in the gap between individual's food-evoked emotions and food choice and buying behavior in the supermarket, such as food culture, habitats, incomes, and family structure in addition to food-evoked emotions. However, finding even more accurate measures or proper combination of measures to better interpret individual's food-evoked emotions is a definite step to better predict individual's actual food choice and buying behavior.

## Author contributions

DK, AT, and A-MB constructed the inclusion criteria, searched the relevant literature, put forward the review idea, read the literature, and organized the article structure. DK wrote the draft manuscript. AT, A-MB, VK, and JvE critically revised the manuscript on article structure, logical organization and language.

### Conflict of interest statement

The authors declare that the research was conducted in the absence of any commercial or financial relationships that could be construed as a potential conflict of interest.
