# Supplementary material for: Methods for Evaluating Emotions Evoked by Food Experiences: A Literature Review
Source: Front Psychol. 2018 Jun 8;9:911. doi: 10.3389/fpsyg.2018.00911 (PMC6002740; doi:10.3389/fpsyg.2018.00911)
Supplement: Supplementary file 2 [file Table_2.docx]

Table S2. Summary of methods used in all included studies on the assessment food elicited emotions and key findings regarding their effectiveness.

| Study | Products | Emotion assessment methods | Key findings regarding methods |
| --- | --- | --- | --- |
| Ares et al., 2010 | - Chocolate milk desserts | - CATA with 3 hedonic terms - 9-point Hedonic Scale | - Hedonic terms did not differ significantly between drinks. - Liking revealed specific preferences. |
| Ares et al., 2008 | - Yogurts | - Word association - Hard laddering | - Word association yielded affective valence of products. - Hard laddering yielded cognitive and evaluative information but no affective information. |
| Awazu, 2013 | - Chocolate, cookies | - 7-point Hedonic scale | Not presented. |
| Barylko-Pikielna et al., 2004 | - Apple and orange juice | - 9-point Hedonic scale - Hedonic ranking | - Both methods correlate strongly for younger people, weaker for elderly. - Hedonic ranking more sensitive to subtle product differences than hedonic rating. - Both methods have the same discriminative power when product differences are moderate. - Hedonic ranking is a simpler and more ‘user friendly’ method. - Both methods yield repeatable results over time. |
| Benson and Scholey, 2014 | - Vodka - Red bull | - POMS, 65 terms on 5 pt scales - VAMS, 16 terms rated on VAS, measuring alertness, calmness, contentedness | - POMS was sensitive to temporal biphasic alcohol effects on mood. - VAMS scores were stable over time. |
| Bhumiratana et al., 2014 | - Coffee | - 86 affective terms on 5-pt scales - 9-pt Hedonic scale | - Products with distinct emotional profiles had similar liking scores. - Liking did not predict product acceptability. |
| Brunyé et al., 2013 | - Images of various foods | - Postural sway - 5-point Hedonic scale | - Participants leaned toward and away from preferred and non-preferred food items. |
| Caporale et al., 2009 | - School lunch | - 7-point Hedonic scale - Amount consumed | - Hedonic ratings predicted food consumption by 4-5 year old children. |
| Cardello et al., 2012 | - Chocolate - Chips | - EsSense Profile (5-pt rating scales) - LAM Hedonic scale | - Food elicited mainly positive emotions (hedonic asymmetry). - Liking correlated highly with emotional valence. |
| Chaya et al., 2015 | - Beers | - 44 affective terms in 12 categories rated on VAS - Liking rated on VAS | - Emotional terms revealed gender effects that were not reflected in the liking ratings. |
| Chung et al., 2012 | - Salad dressings - Beverages | - 9-point Hedonic scales for overall liking, appearance, flavor, and texture - CATA to check reasons for liking | - CATA yielded drivers of (dis)liking. - Familiarity determines liking. |
| Clark, 1998 | - Fruit cakes | - 9-pt Hedonic scale - Preference mapping | - Preference mapping yielded drivers of liking. |
| Collinsworth et al., 2014 | - Orange soda - Dairy beverages - Cheeses | - CATA with 12 affective terms - CATA with 12 predefined affective images - CATA with 12 self-selected affective images - 9-point Hedonic scales for overall, flavor, and texture liking | - CATA with self-selected affective images was more sensitive and yielded more consistent results than words-only CATA in identifying food elicited emotions. - CATA with self-selected affective images was only method sensitive for gender differences. |
| Cordonnier and Delwiche, 2008 | - Lemonades | - Rank rating - 9-pt Hedonic scale | - Rank rating yielded comparable results to hedonic rating. |
| Dalenberg et al., 2014 | - Breakfast drinks | - EsSense Profile (5-pt rating scales) - PrEmo - Liking rated on VAS | - Liking moderately predicts product choice. - Valence strongly predicts product choice. - Valence + liking best predict product choice. - PrEmo better predicts product choice than EsSense Profile. |
| Danner et al., 2014 | - Orange juice | - Recognition of automatic and intentional facial expressions (FaceReader4) - 9-pt Hedonic scale | - Intensity of facial expressions correlated with liking. - Intentional expressions yielded better discrimination. - Automatic (neutral) expressions predicted disliking but not liking. |
| Davies et al., 2012 | - Flavored drinks and solutions | - 9-pt Hedonic scale - Drink pick-up latency | - Pick-up latency appeared an indirect measure for liking. |
| de Wijk et al., 2014 | - Breakfast drinks | - Electrodermal activity (averaged amplitude µSiemens across 10 s after stimulus presentation) - Heart rate (beats per minute) - Skin temperature (the palm of non-dominant finger) - Facial expressions (FaceReader4) - Liking rated on VAS | - Liking did not differ systematically between drinks. - HR, ST and facial expressions differed between drinks. - Liking correlated with neutral facial expressions. |
| de Wijk et al., 2012 | - 3 liked foods - 3 disliked foods | - Electrodermal activity (7s post- minus pre-values in µSiemens) - Heart rate (averaged over the 7s pre- and post-event periods expressed in beats per minute) - Finger temperature (averaged over the 7s pre- and post-event periods) - Facial expressions (FaceReader) | - Facial expressions and SCR correlate with disliking. - Finger temperature was higher for liked than for disliked foods. |
| den Uijl et al., 2016a | - Gingerbreads - Chocolates | - EsSense25 (5-pt rating scales) - 9-pt Hedonic scale | - Reported emotions are age dependent. - Younger people report both valence and arousal. - Older people report mainly valence but less arousal. - Older people give less extreme ratings. |
| den Uijl et al., 2016b | - Gingerbreads - Chocolates | - PrEmo2 - Affect Grid - 9-pt Hedonic scale | - Self-reported emotion of older people is mainly valence driven. - Older people score low on negative emotions. - Liking was age dependent. |
| Desmet and Schifferstein, 2008 | - Sweet bakery snacks - Savory snacks - Pasta meals | - 22 emotion term (5-pt scales) | - Pleasant emotions reported more frequently than unpleasant ones (hedonic asymmetry). |
| di Pellegrino et al., 2011 | - Crackers - Cookies - Biscuits | - Taste pleasantness (11-pt scale) - Taste intensity (11-pt scale) - Visual selective attention | - Changes in pleasantness ratings correlated with changes in attentional bias. |
| Dorado et al., 2016 | - Beers | - 44 terms in 10 beer specific emotion categories (VAS) - Liking (VAS) - Familiarity (VAS) | - Liking independent of scenario. - Scenarios increased familiarity. - More significant differences are observed between emotional responses to more familiar beers. |
| Duka et al., 1998 | - Alcoholic mixture - Non-alcoholic mixture | - Preference (VAS) - Liking (VAS) - Mental states (VAS) - Number and duration of eye fixations - Electrodermal activity (mean values over 1-minute intervals in µSiemens) - Choice behavior - Amount consumed | - Choice behavior correlated with nr fixations. - No relation between preference and choice behavior. - No relation between EDA and choice behavior or amount consumed. - Alcohol sampling increased subjective ratings of mood states. - Amount of alcohol consumed positively correlated with increased subjective ratings. |
| Einöther et al., 2015 | - Tea | - Affect Grid | Not presented. |
| Einöther et al., 2016 | - Tea | - Affect Grid - Affect self-report scale (ASR: 24 terms) - Implicit positive and negative affect test (IPANAT) | - Affect Grid was sensitive for transient and subtle valence changes. - ASR and IPANAT did not register valence changes. - IPANAT sensitive for changes in positive moods but not for negative moods. |
| Esteves et al., 2010 | - IAPS images | - Electrodermal activity (amplitude of maximal deflection between 1 and 4 seconds after stimulus onset) - Valence and Arousal ratings (SAM) - State and Trait Anxiety (STAI) | Not presented. |
| Fairbairn and Sayette, 2013 | - Alcohol - Placebo | - Duchenne smiling duration - Positive and negative affect (PANAS) - 8 mood terms on 6-pt scales | - Autocorrelation of Duchenne smiling predicted self-reported mood. - Negative facial expressions are brief and infrequent. |
| Garcia-Burgos and Zamora, 2013 | - Chocolate drink - Grapefruit juice | - 9-pt Hedonic scale - Facial expressions (FaceReader4) | - Moderate correlation (-0.4) between hedonic rating and facial expression of disgust |
| Geier et al., 2016 | - Water - Milk - Bread - Sugar | - Empathic food test - Multi-Dimensional mood questionnaire - 7-pt Hedonic scale | - The three methods yielded different preferences and effect sizes - The differences are product specific - EFT appeared slightly more discriminative than MDMQ |
| Gil et al., 2009 | - Food and non-food images | - Disgust, Pleasure, Neutrality ratings on 7-pt scales - Valence and Arousal (SAM) - Temporal duration judgement | - Viewing disliked food induces temporal underestimation. - Magnitude of this effect increases with disliking. |
| Grabenhorst et al., 2008 | - MSG (taste only) - MSG with vegetable odor (flavor) | - fMRI (subtraction of BOLD activation produced by tasteless control from those produced by a taste or other stimulus) - 5-pt Hedonic scale (VAS) | - Perceived pleasantness correlated with activity in the orbitofrontal cortex and the pregenual cingulate cortex. - Cognitive factors modulate brain mechanisms representing pleasantness. - Perceived pleasantness correlated with activity in the central striatum when cognitively modulated by word labels. |
| Gutjar et al., 2015a | - Breakfast drinks - Dessert products | - 9-pt Hedonic scale - EsSense Profile (5-pt rating scales) - Food choice | - Product choice better predicted by valence + liking than by liking alone. - Higher liking was strongly related to positive valence. - Liking was not related to arousal. |
| Gutjar et al., 2015b | - Breakfast drinks | - EsSense Profile (5-pt rating scales) - PrEmo - Liking (VAS) - Product choice | - Only few emotions were strongly related to liking. - Most emotions were only weakly related to liking. - Product choice mainly related with (positive) valence. |
| He et al., 2016 | - Fish odor - Orange odor | - Pleasantness (VAS) - PrEmo - Facial expressions (FaceReader4) | - Facial expressions signal both odor valence and intensity. - Facial expressions yield a continuous measure of emotions over time. - Facial expressions distinguish initial and subsequent reactions to a product. - Different emotions have distinct temporary profiles. - Unpleasant odors yield greater facial reactions. - PrEmo yields only odor valence. - PrEmo correlates strongly with facial expression. - Repeated application of PrEmo may trigger negative emotions like boredom. |
| Hebert et al., 2015 | - Appetitive, neutral and disgusting food images | - Postauricular reflex (EMG: template matching on multichannel averaged signals) - Startle reflex (EOG) - Valence and Arousal ratings (SAM) | - Arousal ratings did not differ between neutral and disgusting food images. - Appetitive images were rated as more arousing than neutral or disgusting images. - Startle modulation signals Valence (liking) rather than approach (wanting). - Valence affects SR in a direction opposite to PAR. |
| Hein et al., 2008 | - Breakfast bars | - 9-pt Hedonic scale - VAS - LAM scale - Best-Worst scaling - Hedonic ranking | - Best-worst scaling yielded overall superior discrimination. - Hedonic ranking discriminated products the least. - Best-worst scaling correlated with the largest number of sensory attributes. - Best-Worst Scaling and VAS were judged easiest to use. - Hedonic Ranking was judged most difficult to use. - Best-worst ranking is the most demanding method. - The LAM scale correlated with the least number of sensory attributes. - LAM and VAS yielded similar performance. |
| Hoogeveen et al., 2015 | - Solutions | - 7-pt Hedonic scale - fMRI | - Compared to young people, older people reported higher liking ratings for sweet and salty, lower ratings for sour, and similar ratings for bitter. - This may reflect the reduction of right amygdala activity in older persons. - Age does not affect brain areas mediating taste information. - Older people need less brain activity to integrate taste and somatosensory information. - Older people focus attention less efficiently to taste stimuli. |
| Horska et al., 2016 | - Dry white wines | - EEG for five seconds after stimuli onset (no further details provided). - Facial expressions (FaceReader). - 9-pt Hedonic scale | - Distinct emotions were observed for different products. - Hedonic rating reflects taste perception. |
| Jaeger et al., 2013 | - Different foods | - EsSense Profile 5-pt rating version - EsSense Profile CATA version - 9-pt Hedonic scale - “*How do you feel after having eaten this sample?”* - Most and least applicable of 5-6 affective terms - Free elicitation of emotions | - The EsSense Profile task was judged easy and intuitive. - A total of 20 out of the 25 words of the EsSense Profile were judged to be unrelated to food, making the task weird/odd/unusual. - Some terms of the EsSense Profile were not readily understood, misinterpreted, just too similar to each other, or simply not experienced. - The EsSense Profile task was judged too long and repetitive. - The EsSense Profile task induced biased answering: listed emotions are rated even if they don’t seem to apply, repetitive application causes participants to vary their answers. - Hedonic scaling was judged easy and natural. - The CATA version of the EsSense Profile was not judged as weird, but some terms were misunderstood. - The EsSense Profile may not capture the full range of emotions people may experience in response to food: Free elicitation yielded far less emotion words than used in the EsSense Profile. - Thus, the EsSense Profile probably does not measure emotions. |
| Jaeger and Hedderley, 2013 | - Kiwifruit, apples, chocolates, beverages | - EsSense Profile 5-pt rating version | - Emotional traits affect EsSense Profile ratings more for food word responses than for actual food tasting. - Numerous gender effects on EsSense Profile ratings were observed. |
| Jaeger et al., 2008 | - Minced pork patties | - Hedonic scale on VAS - Best-Worst scaling | - Hedonic rating and BWS are highly correlated. - BWS showed more discriminating power. |
| Jager et al., 2014 | - Dark chocolates | - Temporal dominance of emotions (checking the most dominant out of 10 emotions) - 9-pt Hedonic scale | - TDE yields different temporal emotional profiles for different products. - Temporal emotional and sensory profiles are related. - TDE can only measure a limited number of affective terms. |
| Kim et al., 2013 | - Bottled tea products | - CATA/RGM based FCP - Cued elicitation | - CATA/RGM based FCP and cued elicitation had similar performance. - Cued elicitation more efficient than CATA/RGM based FCP. |
| Kim and O'Mahony, 1998 | - NaCl solution | - PRR - 9-pt Hedonic scale | - PRR has more discriminative power than 9-pt hedonic rating one by one. |
| King and Meiselman, 2010 | - Various foods | - EsSense Profile | - Higher emotional intensities correlated with higher overall acceptability. - Differences in emotional profiles do not predict differences in product acceptance. - Food related self-reports are typically positive (hedonic asymmetry). - Emotional profiles are gender specific. |
| King et al., 2013 | - Spices | - EsSense Profile CATA version - EsSense Profile 5-pt rating version | - CATA more efficient than rating version. - Rating version more sensitive for product differences. - CATA reduces the number of reported emotions. - Terms selected with CATA represent strongest emotions. |
| Kostyra et al., 2016 | - Smoked hams | - Facial expressions (FaceReader4) - 9-pt Hedonic scale | - Emotional expressiveness of consumers was rather small (most had “poker faces”). - Hams predominantly elicited neutral and negative emotions. - Emotions varied over time. - Emotions did not correlate with liking. |
| Kozak and Cliff, 2013 | - Yogurts - Visual appearance of apples | - 9-pt Hedonic scale - Hedonic ranking | - Hedonic ranking introduces false variability. - Hedonic rating is preferred to ranking. |
| Kringelbach et al., 2003 | - Chocolate milk - Tomato juice | - fMRI (contrasting food activated areas with control condition) - Hedonic rating scale (labelled VAS) | - Sensory specific activation in the orbitofrontal cortex correlates with subjective pleasantness. |
| Kuenzel et al., 2011 | - Flavored drinks | - 5-pt Hedonic scale - 9-pt affective scales | Not presented. |
| Kuenzel et al., 2010 | - Flavored drinks | - 5-pt Hedonic scale - Activity (Joy, Contentment) ratings from Intuitive Language Test | - Liking depended on conditioned emotions. - Positive evoked emotions yielded increased liking for moderately liked drinks and decreased liking for highly liked drinks. |
| Kuesten et al., 2014 | - Phytonutrient aromas | - PANAS with 20 terms - I-PANAS-SF with 10 terms - Hedonic scale | - PANAS and I-PANAS-SF yield consistent results. - Both PA and NA yielded higher discrimination between products than overall liking. - PA also discriminates product users. |
| Kwak et al., 2013 | - Rice wines - Soy beverages - Snacks | - 9-pt unidirectional Liking and Disliking scales | - Familiar products evoke stronger bipolar conceptualization. - Unfamiliar products evoke stronger bivariate conceptualization. |
| Labbe et al., 2015 | - Coffee beverages | - Hedonic scale - 39 Emotional terms - Importance of each of the 5 senses - Satisfaction from each of the 5 senses   all rated on VAS | - Dynamics of sensory importance differed between senses. - Consumer motivation (enjoyment vs stimulation) affected pleasantness and experienced emotions. |
| Lange et al., 2002 | - Champagne | - Experimental (Vickrey) auction - Hedonic rating scale (labelled VAS) | - Bids and hedonic ratings yielded the same product ranking. - Bids were more discriminative than hedonic rating. - Hedonic rating sensitive for the intrinsic value of a product. - Bids sensitive for the extrinsic value of a product. |
| Leitch et al., 2015 | - Sweetened tea solutions | - 9-pt Hedonic scale - Facial expression response (FaceReader5) - EsSense Profile CATA version | - Facial expressions did not discriminate between products. - No correlation between facial expressions and hedonic scores. - Temporal profiles of facial expressions were more robust in showing discriminating products. - CATA showed that liked products were associated more with neutral to positive emotions, while disliked products were associated more with neutral to negative emotions. |
| Lévy and Köster, 1999 | - Soft drinks - Alcoholic beverages | - 9-pt Hedonic scale - Liking rated on bipolar VAS - Product choice | - Product preferences changed over repeated exposure (both within and between sessions). - Product choice was a better predictor of preference than initial hedonic judgements. |
| Lim and Padmanabhan, 2013 | - Vegetables, taste and smell | - Labeled hedonic scale (LHS) | - Odor liking correlated significantly with taste liking. - Hence, odor liking can be a predictor of taste liking. |
| Macht et al., 2003 | - Low-, medium- and high-energy foods | - 7 affective terms rated on 7-pt unipolar scales - 5-pt Hedonic scale | - Hedonic ratings did not discriminate among foods. - High-energy food evoked more negative and less positive emotional associations than medium- and low-energy foods. |
| Manzocco et al., 2013 | - Images of fruit salads of different quality | - 29 affective attributes - 9-pt Hedonic scale | - Positive emotions were reported more frequently than negative emotions (hedonic asymmetry) - Strong correlation between visual quality indicators (liking, browning) and some specific emotions. |
| Marczinski et al., 2014 | - Energy drinks | - POMS - Cued go/no-go task - Systolic and diastolic blood pressure - Pulse rate | - Consumption of energy drinks - improved subjective states, - had no effect on go-no-go task performance, - elevated blood pressure, - had no effect on heart rates. |
| McClure et al., 2004 | - Coca-Cola - Pepsi | - fMRI - Behavioral preferences - Stated preferences | - No correlation between stated and behavioral preferences. - For blind tasting relative activity in the ventromedial prefrontal cortex predicts behavioral preferences. - Brand information significantly influenced both behavioral preferences and brain activity. |
| Mielby et al., 2012 | - Fruit- and vegetable- based snacks | - Expected Liking rated on 7-pt facial rating scale - Actual Liking rated on 7-pt facial rating scale | - Expected liking was significantly higher than actual liking for non-surprising snacks. - Actual liking was significantly higher than actual liking for surprising snacks (containing nonvisible food compounds). |
| Mojet et al., 2015 | - Yogurts | - Actual liking - Expected liking - Emotive Projection Test - Autobiographical Congruency Test - Visual attention - Facial expression response | - VA had no significant correlation with either liking but did not differentiate between products. - ACT did not differentiate between products and was found unfit for use in product research. - EPT was the most promising measure, since it had no significant correlation with either liking and differentiated between products. |
| Ng et al., 2013 | - Blackcurrant squashes | - 9-pt Hedonic scale - EsSense Profile 5-pt rating version - Consumer defined emotion lexicon (CD-CATA) | - High correlation between positive and unclassified EsSense emotions and liking. - EsSense profile discriminated products with similar liking scores. - CD-CATA responses showed hedonic asymmetry. - High correlation between CD-CATA checked emotions and liking. - CD-CATA terms discriminated more between products than liking, and more so than the EsSense Profile. - CD-CATA had more balanced positive and negative terms. - CATA process was found easier and more natural. - Conclusion: emotional terms are complementary to liking. |
| O'Doherty et al., 2001 | - Glucose - Saline | - fMRI (MEDx: changes in voxel intensity between taste stimulus and tasteless control) - 5-pt Hedonic scale | - Pleasant and unpleasant tastes both activated the orbitofrontal cortex and the amygdala. |
| Parma et al., 2014 | - Cake | - 9-pt Hedonic scale - Reach-to-grasp kinematics | - Significant negative correlation between liking and time at which wrist reached maximum velocity. - Kinematics may serve as an implicit affective product evaluation measure. |
| Piqueras-Fiszman et al., 2014 | - Food images | - Approach-Avoidance reaction time (push vs pull on joystick) - Liking on VAS - Disgust on VAS - Valence on VAS - Arousal on VAS | - Significant interaction between food valence and direction of joystick movement. - Liking discriminated between groups with different hunger state for positive and neutral images. - Implicit measure discriminated between groups with different hunger state for negative images. - Valence strongly correlated with liking (positively) and disgust (negatively). |
| Plassmann et al., 2008 | - Wines | - fMRI (BOLD contrast) - 6-pt Hedonic scale | - Pleasantness ratings correlated with mOFC activity. |
| Poole et al., 2007 | - Mandarins | - Experimental auction - 7-pt Hedonic scale | - Bid behavior (willingness to pay) closely reflected hedonic rating. - Hedonic rating much simpler to implement than auction procedure. |
| Porcherot et al., 2015 | - Kir aperitifs with different fruits | - ScentMove - VAMS - Liking rated on VAS | - ScentMove failed to differentiate short term emotional responses between products. - VAMS measured significant mood changes for one of the products. - Liking did not discriminate between products. |
| Resano et al., 2009 | - Cured ham | - Liking rated on VAS - Purchase behavior (scanner data) - Preference mapping | - Preference mapping identified distinct consumer segments with consistently different purchasing behavior. |
| Rosas‐Nexticapa et al., 2005 | - Strawberry flavored yogurts | - 9-pt Hedonic scale - Hedonic ranking - 5-pt likelihood-to-buy scale - Likelihood-to-buy ranking - Purchase frequency | - Hedonic ratings and likelihood-to-buy ratings both predicted purchase frequency. - Hedonic ranking and likelihood-to-buy ranking did not predict purchase frequency. |
| Schifferstein et al., 2013 | - Buying, preparing and eating dehydrated food products | - 12 emotion terms rated on 3-pt scales - Importance of each sensory modality on 5-pt scales | - Emotional responses affected both by actual perception and pre-existing attitudes and beliefs. - Positive and negative emotions are independent factors. - Emotional responses unrelated to perceived importance of the different senses. |
| Schouteten et al., 2015 | - Crips, chocolate, cola, burgers, vanilla pudding | - EmoSensory Wheel 5-pt RATA version with 14 or 17 emotional terms - 9-pt Hedonic scale | - The EmoSensory Wheel could discriminate equally liked products, even when their overall liking was low. |
| Schouteten et al., 2016 | - Insect-, plant- and meat-based burgers | - EmoSensory Wheel 5-pt RATA version with 14 emotional terms - 9-pt Hedonic scale | - Information about the nature of the food affected hedonic ratings more than emotional conceptualizations. |
| Schutz and Cardello, 2001 | - Lasagna, chocolate chip cookies, prunes, tomato juice, diet cola, crackers | - 9-pt Hedonic scale - Labelled Affective Magnitude scale | - LAM has equal reliability and sensitivity to hedonic scale. - LAM has somewhat greater discrimination among highly liked foods than hedonic scale. |
| Silva et al., 2016 | - Tasting beer, wine and non-alcoholic beer - Viewing images of consumption situations | - Free association of emotional words - Association of faces showing emotions to products | - Emotional association revealed different conceptualization between (alcoholic and non-alcoholic) beverages. |
| Small et al., 2001 | - Chocolate | - PET scans (regional Cerebral Blood Flow (rCBF): comparison between test stimuli and water as a control) - 20-pt Hedonic scale | - Sensory and limbic processing of taste are integrated. - Different neural substrates mediating positive/appetitive and negative/aversive stimuli. |
| Spinelli et al., 2014 | - Chocolate and hazelnut spreads | - EmoSemio on 5-pt scale - EsSense Profile on 5-pt scale - 9-pt Hedonic Scale | - EmoSemio and EsSense Profile include different emotions. - The use of full sentences in the EmoSemio reduced ambiguity compared to the use of adjectives in EsSense Profile. - Product specific EmoSemio discriminated emotions better than EsSense Profile. - Some items in EsSense Profile discriminated moods, attitudes, personality traits and interpersonal stances better than EmoSemio. - Liking correlated positively with positive emotions and negatively with negative emotions. - EsSense Profile yielded similar emotional profiles for products with significantly different sensory profiles and Liking. - EmoSemio yielded distinct emotional profiles for these products. - Emotion ratings discriminated between products with similar Liking. |
| Spinelli et al., 2015 | - Hazelnut and cocoa spreads | - EmoSemio on 5-pt scale - 9-pt Hedonic scale | - Differences in emotional profiles result from sensory differences. - Liking correlated with emotions. - Emotions highly determined by expectations. - Familiarity influences emotions. - Products with different likings had few but significant differences in emotional profiles. |
| Sudre et al., 2012 | - Wheat flakes cereals | - 7-pt Hedonic scale used in 3 different ways:   - once, for overall Liking,   - at 4 specific moments during mastication, or   - for initial response followed by responses to changes only. | - Overall liking correlated with dynamic liking at the start of the mastication period. - Individuals have distinct dynamic liking profiles |
| Sulmont-Rossé et al., 2008 | - Fruit drinks | - Liking rated on VAS | - Liking correlated negatively with Familiarity. - (dis-)Liking of unfamiliar drinks (de-)increased with exposure. - Liking of familiar drinks did not vary with exposure. |
| Swan et al., 2013 | - Sweet, sour and tasty flavor sprays - Neutral IAPS images | - SAM Pleasantness and Arousal on 9-pt scales | - Participants tasting sweet (sour and spicy) flavor rated neutral images as more (less) pleasant. - Arousal ratings did not differ between flavor conditions. |
| Symoneaux et al., 2012 | - Golden apples | - 7-pt Hedonic scale - Open comments on Likes and Dislikes | - Hedonic preferences correlated with preferences derived from open comments. - Open comments yielded more information on the drivers of liking. |
| Thomson et al., 2010 | - Dark chocolates | - List of 24 affective adjectives - Best-Worst scaling of lexicon terms | - BWSLT effectively discriminates between different products. - BWSLT yields emotional conceptualizations that link to sensory product characteristics. |
| Tsourides et al., 2016 | - Food/non-food images | - MEG (machine learning pattern classification analyses and conventional evoked response field (ERF) analyses) - Hedonic rating (no details given) | - No neural correlates for food Liking. |
| Tuorila et al., 2015 | - 36 different meals | - 7-pt Hedonic scale | Not presented. |
| Varela et al., 2014 | - Instant coffees | - 9-pt Hedonic scale - Hedonic ranking with open comments on Likes and Dislikes | - Hedonic Scale and Hedonic Ranking gave same ordering. - Hedonic Ranking is a quick and easy (intuitive) method. - Open comments on likes and dislikes identified consumer segments and drivers of liking. - Open comments yield more information (are less restricted) than CATA. |
| Villanueva et al., 2000 | - Commercial candies | - 9-pt Hedonic scale - Self-Adjusting scale (VAS) - Hedonic ranking | - 9-pt Hedonic Scale produced a slight deviation from normality and a lack of homoscedasticity. - Self-Adjusting Scale produced homogeneous variances but large deviations from normality, resulting in the lowest discriminating power. - Hedonic Ranking showed the highest discriminating power |
| Villanueva et al., 2005 | - Orange juices | - Hybrid Hedonic scale - 9-pt Hedonic scale - Self-adjusting scale - Hedonic ranking | - Hybrid Hedonic Scale has the best discriminative power, complies with normality and homoscedasticity, and is considered easiest to use by assessors. - 9-pt Hedonic Scale produces unequal sample variances. - Self-Adjusting Scale produced large deviations from normality. - Hedonic Ranking Scale has the lowest discriminative power. |
| Walla et al., 2010 | - Ice cream, yogurt, chocolate, soft drink | - Startle response modulation (EMG: average signal over a window from 35-135 ms after the onset of the acoustic stimulus) - EEG (mean voltage across 250 ms epochs from 1.5 s to 7 s after stimulus onset) | - Startle Response modulation measured motivational state related to product intake. - EEG signals did not discriminate between different products. |
| Weiss et al., 2010 | - Chocolates | - Self-reported Liking, Choosing and Buying Preferences - Take away behavior | - Low correspondence between self-reported liking, choosing or buying preferences and what consumers actually took away. |
| Wichchukit and O’Mahony, 2010 | - Potato chips | - Paired preference test of Liking - Buying preference | - Low correspondence between self-reported Liking and buying preferences and what consumers actually took away. |
| Yeomans and Symes, 1999 | - Macaroni with 2 different cheese sauces | - Hedonic rating on VAS - Amount consumed | - Liking deceased across a meal. - Differences in liking correlated with differences in intake. |
| Yoshimura et al., 2011 | - Shortcakes consumed with virtual audio-visual scenes of tearoom and construction work | - 11-pt scales for rating Pleasantness, Deliciousness and Scene-consonance - EEG (contrasting the mean occupancy rates of theta, alpha1, alpha2, alpha3, and beta frequency bands for 180 seconds for tearoom and construction work scenes) | - Pleasantness discriminated between scenes. - Occupancy rate of beta frequency band correlated positively with deliciousness. |
| Zandstra et al., 2000 | - Sandwiches | - 10-pt Hedonic scale - Amount consumed | - Relation between food intake and pleasantness changes over time - Pleasantness is constant over repeated exposure - Food intake of less-preferred foods increased with repeated exposure |
| Zandstra et al., 1999 | - Yogurts | - 10-pt Hedonic scale with faces - Amount consumed | - Individual pleasantness ratings correlated positively with ad libitum consumption. |
| Zeinstra et al., 2009 | - Liquids with different tastes | - Hedonic ranking - Facial expression response | - Facial expressions signaled disliking but not liking. |
